# Thin-section computed tomography-determined usual interstitial pneumonia pattern affects the decision-making process for resection in newly diagnosed lung cancer patients: a retrospective study

**DOI:** 10.1186/s12890-017-0565-5

**Published:** 2018-01-05

**Authors:** Naozumi Hashimoto, Akira Ando, Shingo Iwano, Koji Sakamoto, Shotaro Okachi, Asuka Matsuzaki, Yu Okada, Kenji Wakai, Kohei Yokoi, Yoshinori Hasegawa

**Affiliations:** 1 Department of Respiratory Medicine, Nagoya University Graduate School of Medicine, 65 Tsurumai-cho, Showa-ku, Nagoya, 466-8550 Japan; 2 Department of Radiology, Nagoya University Graduate School of Medicine, Nagoya, Japan; 3 Department of Preventive Medicine, Nagoya University Graduate School of Medicine, Nagoya, Japan; 4 Department of Thoracic Surgery, Nagoya University Graduate School of Medicine, Nagoya, Japan

**Keywords:** Bronchoscopy, CPFE, Decision-making process, Thin-section CT, Thoracic surgery

## Abstract

**Background:**

There is only limited information on the impact of thin-section computed tomography (TSCT)-determined usual interstitial pneumonia (UIP) pattern in the decision-making for resection in newly diagnosed lung cancer patients.

**Methods:**

In this retrospective analysis, data were reviewed from 499 newly diagnosed lung cancer patients who received bronchoscopy between 2010 and 2014. The clinical impact of TSCT-determined UIP pattern on the decision-making process for resection in this cohort was evaluated.

**Results:**

The prevalence rate of TSCT-determined fibrosis was 14.8% (74/499 cases), 86.5% (64/74 cases) of which also had TSCT-determined emphysema. The fibrosis group comprised 40 patients with possible UIP and 34 patients with the UIP pattern. Among surgical candidates, the number of surgeries performed was lower in the fibrosis group (60.8%) than in the normal and emphysema groups (84.7 and 77.3%, respectively). Although the proportion of possible UIP did not differ between surgical candidates and patients with resected lung cancer, the proportion of UIP pattern in patients with resected lung cancer was decreased by 8.5%, compared to the surgical candidates. Although measurement of diffusing capacity of the lung for carbon monoxide (DLCO) was performed in more than 97% of patients with thoracic surgery, only 58% of patients without thoracic surgery had DLCO measurement. Multivariate analysis showed that the finding of UIP pattern independently affects the decision-making process for thoracic surgery. The adjusted odds ratios for the comparison between the patients without fibrosis and the patients with UIP pattern was 0.266 (95% confidence intervals: 0.087–0.812).

**Conclusions:**

The presence of TSCT-determined UIP pattern might independently affect the decision-making process for proposing thoracic surgery with curative intent.

## Background

There are increasing numbers of elderly with smoking history as well as lung cancer patients with chronic lung diseases such as chronic obstructive lung disease (COPD) and pulmonary fibrosis. Many studies suggest that spirometry-determined airflow obstruction is related to worse postoperative outcomes among resected lung cancer cases [1–5]. Therefore, severe airflow obstruction might independently affect the decision-making process for proposing thoracic surgery [6].

Chest thin-section computed tomography (TSCT) with a slice thickness of less than 2 mm has been commonly used for the qualitative and quantitative evaluation of pulmonary emphysema and/or fibrosis [7]. There is increasing awareness that the presence of emphysema or fibrosis clinically affects postoperative and survival outcomes for patients with resected lung cancer [8–11]. TSCT-determined usual interstitial pneumonia (UIP) pattern is a characteristic finding of idiopathic pulmonary fibrosis (IPF) which has a death rate worse than that of many cancers [12, 13]. In patients with resected lung cancer, acute exacerbation of interstitial lung diseases might be associated with the finding of TSCT-determined UIP pattern [9]. Although we recently demonstrated that the presence of TSCT-determined combined pulmonary fibrosis and emphysema (CPFE) yielded worse postoperative and survival outcomes in patients with resected lung cancer, less than 30% of patients with fibrosis had TSCT-determined UIP pattern among resected lung cancer cases [14]. Therefore, we hypothesized that TSCT-determined UIP pattern might affect the decision-making process for resection in newly diagnosed lung cancer patients. New therapeutic options for stable chronic lung diseases such as COPD/emphysema and pulmonary fibrosis have been utilized in the worldwide [15, 16]. To establish appropriate therapeutic strategy for chronic lung diseases among newly diagnosed lung cancer patients, clinical impact of TSCT-determined UIP pattern on the decision-making process for proposing thoracic surgery should be determined. Bronchoscopy is performed for most lung cancer patients before the lung cancer treatment decision is made [17, 18].

In the present study, we determined the clinical impact of TSCT-determined UIP pattern on the decision-making process for resection among 499 newly diagnosed lung cancer patients who received bronchoscopy.

## Methods

### Population

Patients with newly diagnosed lung cancer who underwent bronchoscopy at Nagoya University Hospital from January 2010 and December 2014 were the subjects of this retrospective study. The study was approved by the Institutional Review Board of Nagoya University Graduate School of Medicine (No 2014–0052-2).

### Collection of data on patient characteristics

Information about patient characteristics, pathological diagnosis of lung cancers, and clinical staging of lung cancers, was obtained from hospital records as previously reported. Spirometry screening assessment was routinely performed when patients admitted to hospital for bronchoscopy [6]. Diffusing capacity of the lung for carbon monoxide (DLCO) was measured using the single breath-holding method and the Burrows’ equation was utilized for DLCO [2]. Subjects were assigned to the COPD group, if they had airflow obstruction as determined by an FEV1/FVC ratio below 0.70 [6]. Severity of airflow limitation in COPD was determined according to the Global Initiative for Chronic Obstructive Lung Disease (GOLD) grade [6, 19]. Clinical staging of lung cancer was based on the tumor, node, and metastasis (TNM) staging using the standards of the Union International Contre le Cancer (UICC), 7th edition [20]. Histological type of adenocarcinoma, squamous cell carcinoma (Sq), large cell carcinoma (Large), small cell lung carcinoma (SCLC), and non-small cell lung carcinoma (NSCLC), and carcinoid, were determined according to the World Health Organization’s classification [21].

### Assessment of emphysema and fibrosis on chest CT

Involvement of emphysema and fibrosis was evaluated by TSCT as previously reported [14]. Axial TSCT images of the whole lung with a slice thickness of 0.5–1.0-mm were reconstructed at the same increment by using a high-spatial frequency algorithm. After the initial interpretation by at least two radiologists, the evaluation was performed by a chest radiologist (SI) who had 20 years of experiences in reading thoracic CTs. For the present study, emphysema and fibrosis on chest TSCT were evaluated in accordance with the definition described by Cottin et al. [14, 22]. The severity of emphysema was also evaluated visually according to the Goddard classification as previously reported [14, 23]. The severity of fibrosis was classified visually into three categories as follows: 0, normal; 1, possible usual interstitial pneumonia (UIP) pattern and 2, UIP pattern [13, 14].

### Statistical analysis

All data were checked for completeness, and the analyzed variables were tested for normality of distribution by the Shapiro-Wilk test. One-way analysis of variance (ANOVA) was used to compare normally distributed variables among the groups, and Kruskal-Wallis test was used to compare non-normally distributed variables. The t-test was used to compare normally distributed variables between the surgery and the non-surgery group subjects, and Mann-Whitney test was used to compare non-normally distributed variables. Comparisons of proportions among the groups were made using the χ^2^ test or Fisher exact test. Multivariable logistic regression models were prepared to estimate the decision-making factors for proposing thoracic surgery. Inclusion of variables in the models was based on existing knowledge of the factors for proposing thoracic surgery, including age, histology, severity of airflow obstruction, clinical stage, and severity of fibrosis [6]. Statistical analyses were performed with PASW Statistics version 24.0 software (SPSS Inc., Chicago, IL), and a two-sided *P* value less than 0.05 was considered statistically significant.

## Results

### Demographic distribution of patient characteristics

Data from 596 patients with lung cancer who were sequentially registered and underwent bronchoscopy from August 2010 to July 2014 were obtained from hospital records. The study population excluded 72 patients who had not undergone pulmonary assessment by spirometry. Twenty-five patients (not evaluated by TSCT for the presence of emphysema and/or fibrosis, or who had received repetitive bronchoscopy) were also excluded from the study population. As a result, the study cohort comprised 499 patients (83.7%). In total, 135 (27.1%) patients showed emphysema without fibrosis. Overall, 74 (14.8%) patients had a finding of fibrosis, and 64 (86.5%) of these patients also had emphysema. The remaining 290 (58.1%) subjects showed evidence of neither emphysema nor fibrosis in their chest CT examinations. Although CPFE might be considered by some as an entity that is distinct from either emphysema or fibrosis [24], patients with fibrosis and CPFE were combined into a fibrosis group for further analysis in this study. The characteristics of the 499 study patients are presented in Table 1. The mean age was 70.0 years. Overall, 348 patients were male and 375 had a history of smoking. Compared with the normal group, the emphysema and fibrosis groups were predominantly male and with a higher smoking history. Although more than 65% of the emphysema group had airflow obstruction (determined by an FEV1/FVC ratio below 0.7), less than 40% of the fibrosis group including patients with CPFE had significant airflow obstruction. The severity of emphysema in the fibrosis group was similar to that in the emphysema group and the proportion of emphysema grade 1 was significantly higher than those with emphysema grade 2 or 3. More than 45% of the fibrosis group had grade 2 disease (UIP pattern).

**Table 1:** Patient Characteristics

	All cases (*n* = 499)	Normal (*n* = 290)	Emphysema (*n* = 135)	Fibrosis (*n* = 74)	*P* value
Age, years^a^	70.0 (38–88)	68.4 (38–88)	69.9 (47–86)	71.9 (58–87)	0.012
Gender, male	69.7 (348)	55.5 (161)	88.1 (119)	91.9 (68)	0.001
History of smoking	75.2 (375)	58.6 (170)	99.3 (134)	95.9 (71)	0.001
Diabetes	20.4 (102)	17.2 (50)	22.2 (30)	29.7 (22)	0.049
Ischemic cardiac disease	7.8 (39)	4.8 (14)	11.9 (16)	12.2 (9)	0.014
Spirometric variables					
VC (%)^b^	105.8 (20.3)	109.3 (20.6)	108.4 (19.2)	104.9 (17.8)	0.208
%FEV1 predicted^b^	101.0 (24.2)	105.3 (24.4)	95.0 (22.0)	104.7 (17.6)	0.001
FEV1/FVC below 0.7	46.9 (234)	39.3 (114)	68.1 (92)	37.8 (28)	0.001
Severity of airway obstruction					0.001
Non-COPD	53.1 (265)	60.7 (176)	31.9 (43)	62.2 (46)	
GOLD grade 1	32.3 (161)	26.6 (77)	44.4 (60)	32.4 (24)	
GOLD grade 2	12.6 (63)	10.0 (29)	22.2 (30)	5.4 (4)	
GOLD grade 3	2.0 (10)	2.8 (8)	1.5 (2)	0 (0)	
GOLD grade 4	0 (0)	0 (0)	0 (0)	0 (0)	
TSCT emphysema					
Grade 0	60.1 (300)	100 (290)	0 (0)	13.5 (10)	
Grade 1	26.9 (134)	0 (0)	62.2 (84)	67.6 (50)	
Grade 2	10.2(51)	0 (0)	28.9 (39)	16.2 (12)	
Grade 3	2.8 (14)	0 (0)	8.9 (12)	2.7 (2)	
TSCT fibrosis					
Grade 0	85.2 (425)	100 (290)	100 (135)	0 (0)	
Grade 1	8.0 (40)	0 (0)	0 (0)	54.1 (40)	
Grade 2	6.8 (34)	0 (0)	0 (0)	45.9 (34)	

n indicates number. ^a^Data are shown as mean (range). ^b^Data are shown as mean (standard deviation). All other data are shown as % (number)

### Tumor characteristics

Overall, 211 patients had pathological stage I disease, 75 patients had stage II disease, and 182 patients had stage III or IV disease. The prevalence of adenocarcinoma, squamous cell carcinoma, and other type of histology were 58.1, 25.7, and 16.2%, respectively. To evaluate the association of TSCT-determined emphysema and/or fibrosis with characteristics of lung cancer, pathological stage and histology were compared among the groups (Table 2). The proportion of patients with stage I disease was significantly higher in the normal group and lower in the emphysema and fibrosis groups. Although determining the clinical stage should be essential before proposing the therapeutic options for lung cancer, uncompleted clinical staging did not differ among the groups. Regarding the tumor histology, the prevalence of adenocarcinoma was significantly higher in the normal group, whereas squamous cell carcinoma was significantly higher in the emphysema and fibrosis groups.

**Table 2:** Tumor characteristics

	All cases (*n* = 499)	Normal (*n* = 290)	Emphysema (*n* = 135)	Fibrosis (*n* = 74)	*P* value
Clinical stage					0.053
I	42.3 (211)	47.6 (138)	36.3 (49)	32.4 (24)	
II	15.0 (75)	15.1 (44)	14.1 (19)	16.2 (12)	
III	17.8 (89)	12.8 (37)	24.4 (33)	25.7 (19)	
IV	18.6 (93)	17.9 (52)	19.3 (26)	20.3 (15)	
ND^a^	6.2 (31)	6.6 (19)	5.9 (8)	5.4 (4)	
Histology					0.001
Adenocarcinoma	58.1 (290)	68.6 (199)	46.7 (63)	37.8 (28)	
Squamous cell carcinoma	25.7 (128)	19.7 (57)	34.1 (46)	33.8 (25)	
Others^b^	16.2 (81)	11.7 (34)	19.3 (26)	28.4 (21)	
Thoracic surgery					0.002
yes	55.9 (279)	62.3 (180)	51.1 (69)	40.5 (30)	

n indicates number. All data are shown as % (number)

^a^ND includes not determined

^b^Others include Large, SCLC, NSCLC and carcinoid

### Critical impact of the severity of TSCT-determined fibrosis on the decision to propose thoracic surgery with curative intent

**Fig. 1:**
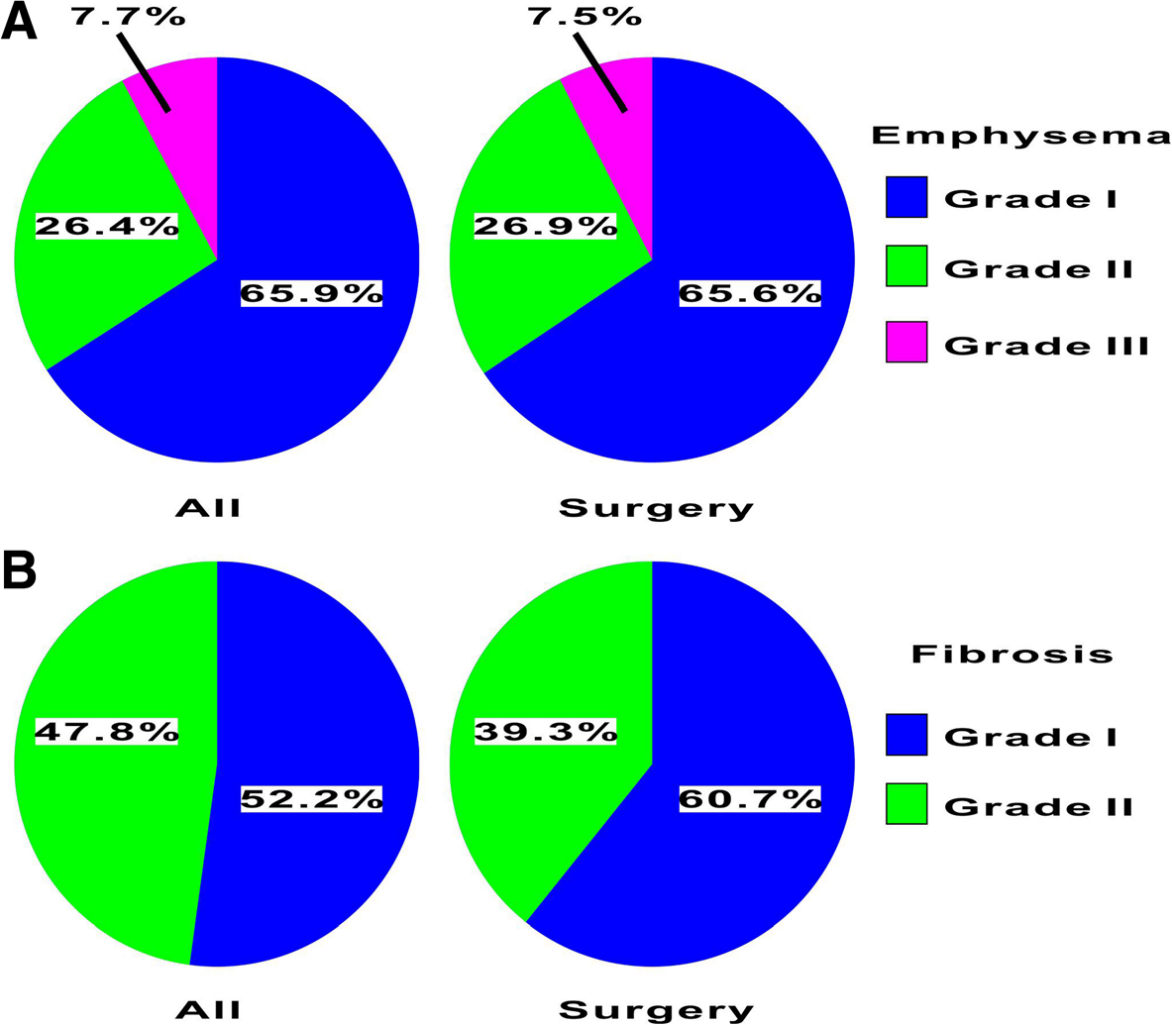
Population of severity of emphysema and fibrosis. **a** The left panel shows emphysema severity among emphysema patients awaiting thoracic surgery with curative intent. The right panel shows emphysema severity in emphysema patients who received thoracic surgery. Blue, emphysema grade 1; green, emphysema grade 2; pink, emphysema grade 3. **b** The left panel shows fibrosis severity among fibrosis patients awaiting thoracic surgery with curative intent. The right panel shows fibrosis severity among fibrosis patients who received thoracic surgery. Blue, fibrosis grade 1; green, fibrosis grade 2

To explore whether the severity of TSCT-determined fibrosis might affect the decision to propose thoracic surgery with curative intent, patients at stage IIIB and IV were excluded from the analysis because they were not eligible for thoracic surgery. In addition, the patients with incomplete clinical staging or SCLC were also excluded. Overall, we evaluated data from 343 patients with lung cancer at stage IA to IIIA who underwent TSCT examination, spirometry, and bronchoscopy. These patients were subdivided according to whether they received thoracic surgery (273 cases) or not (70 cases). The characteristics, spirometric variables, and TSCT data for the patients with or without thoracic surgery are summarized in Table 3. The existence of comorbidities including ischemic disease did not differ among the group. Although measurement of DLCO was performed in more than 97% of patients with thoracic surgery, only 58% of patients without thoracic surgery had DLCO measurement. Even among these surgical candidates, the number of surgeries performed was significantly lower in the fibrosis group (60.8%, 28/46 cases) than in the normal and emphysema groups (84.7%, 177/209 cases; 77.3%, 68/88 cases, respectively). More than 70% of patients with each emphysema grade received thoracic surgery (emphysema grade 1, 61/85 cases; grade 2, 25/34 cases; grade 3, 7/10 cases, respectively) (Table 3 and Fig. 1a). As a consequence, the proportion of each emphysema grade did not differ between surgical candidates and patients with resected lung cancer (Fig. 1a). Although more than 70% of patients with fibrosis grade 1 received thoracic surgery (17/24 cases), only 50% of patients with fibrosis grade 2 received it (11/22 cases) (Table 3 and Fig. 1b). Therefore, among patients with fibrosis and resected lung cancer, those with fibrosis grade 2 were decreased by 8.5%, compared to the surgical candidates (Fig. 1b). Less than 50% of patients with stage III disease received thoracic surgery. More than 80% of patients with adenocarcinoma received thoracic surgery, but more than 30% of patients with squamous cell carcinoma did not. We evaluated the decision-making factors for proposing thoracic surgery (Table 4). Although there were no significant relative odds ratios (ORs) for the comparison between the patients with fibrosis grade 0 and grade 1, the relative ORs for the comparison between the patients with fibrosis grade 0 and grade 2 showed significant confidence intervals (CIs). When the relative ORs for the comparison between the patients with fibrosis grade 0 and grade 2 were adjusted for age, histology, clinical stage, and airflow obstruction, multivariate analysis showed a significant relative OR value of 0.266 (95% CI: 0.087–0.812) (Table 4). Age, GOLD grade 3 airflow obstruction, and clinical stage III-but not squamous cell carcinoma- remained to be independent decision-making factors for thoracic surgery after the adjustment (Table 4).

**Table 3:** Patient and tumor characteristics in the thoracic surgery and non-thoracic surgery groups

	all cases (*n* = 343)	non-surgery (*n* = 70)	surgery (*n* = 273)	*p* value
Age, years^a^	69.8 (38–88)	70.7 (50–86)	68.8 (38–88)	0.001
Gender, male	68.0 (233)	75.7 (53)	65.9 (180)	0.118
History of smoking	72.3 (248)	80.0 (56)	70.3 (192)	0.107
Diabetes	21.0 (72)	28.6 (20)	19.0 (52)	0.810
Ischemic disease	7.9 (27)	12.9 (9)	6.6 (18)	0.083
Spirometric variables				
VC^b^	109.0 (20.0)	101.8 (25.3)	111.2 (19.0)	0.001
%FEV1 predicted^b^	104.1 (23.5)	94.2 (24.7)	106.2 (22.6)	0.011
FEV1/FVC below 0.7	43.7 (150)	50 (35)	42.1 (115)	0.236
DLCO, performed	89.8 (308)	58.6 (41)	97.8 (267)	0.001
DLCO	108.0 (28.8)	96.3 (34.7)	109.9 (27.5)	0.021
Severity of airway obstruction				0.049
Non-COPD	56.3 (193)	50.0 (35)	57.9 (158)	
GOLD grade 1	32.9 (113)	37.1 (26)	31.9 (87)	
GOLD grade 2	9.0 (31)	7.2 (5)	9.5 (26)	
GOLD grade 3	1.7 (6)	5.7 (4)	0.7 (2)	
GOLD grade 4	0 (0)	0 (0)	0 (0)	
Thin section CT				0.001
Normal	60.9 (209)	45.7 (32)	64.8 (177)	
Emphysema	25.7 (88)	28.6 (20)	24.9 (68)	
Fibrosis	13.4 (46)	25.7 (18)	10.9 (28)	
Severity of emphysema				0.065
Grade 0	62.4 (214)	48.6 (34)	65.9 (180)	
Grade 1	24.8 (85)	34.3 (24)	22.3 (61)	
Grade 2	9.9 (34)	12.8 (9)	9.2 (25)	
Grade 3	2.9 (10)	4.3 (3)	2.6 (7)	
Severity of fibrosis				0.002
Grade 0	86.6 (297)	74.3 (52)	89.7 (245)	
Grade 1	7.0 (24)	10.0 (7)	6.2 (17)	
Grade 2	6.4 (22)	15.7 (11)	4.0 (11)	
Clinical stage				0.001
I	60.6 (208)	37.1 (26)	66.7 (182)	
II	21.3 (73)	15.7 (11)	22.7 (62)	
III	18.1 (62)	47.1 (33)	10.6 (29)	
Histology				0.006
Adenocarcinoma	62.7 (215)	48.6 (34)	66.3 (181)	
Squamous cell carcinoma	28.9 (99)	44.3 (31)	24.9 (68)	
Others^c^	8.5 (29)	7.1 (5)	8.8 (24)	

n indicates number. ^a^Data are shown as mean (range). ^b^Data are shown as mean (standard deviation). ^c^Others include Large, SCLC, NSCLC and carcinoid. All other data are shown as % (number)

**Table 4:** Univariate and multivariate analysis of independent factors in decision-making process for proposing thoracic surgery with cure intent

Variables	Unadjusted Odds Ratio	95% CI	P value	Adjusted Odds Ratio	95% CI	P value
Age (per year)	0.927	0.893–0.996	0.001	0.883	0.840–0.927	0.001
Histology						
Adenocarcinoma	reference			reference		
Squamous cell carcinoma	0.412	0.235–0.722	0.020	0.589	0.287–1.207	0.148
Others^a^	0.902	0.322–2.528	0.844	2.258	0.565–9.027	0.249
Severity of airflow obstruction						
Non-COPD	reference			reference		
GOLD grade 1	0.741	0.419–1.312	0.304	1.056	0.516–2.160	0.882
GOLD grade2	1.152	0.413–3.210	0.787	1.500	0.428–5.259	0.527
GOLD grade 3	0.111	0.020–0.629	0.013	0.049	0.007–0.356	0.003
Clinical stage						
I	reference			reference		
II	0.805	0.376–1.724	0.577	0.765	0.318–1.842	0.550
III	0.126	0.066–0.240	0.001	0.066	0.029–0.153	0.001
Severity of fibrosis						
Grade 0	reference			reference		
Grade 1	0.515	0.203–1.306	0.162	0.618	0.201–1.899	0.400
Grade 2	0.212	0.087–0.516	0.001	0.266	0.087–0.812	0.020

^a^Others include Large, NSCLC, and carcinoid

## Discussion

This is the first study showing how the finding of TSCT-determined UIP pattern affects the decision-making process for proposing resection among newly diagnosed lung cancer patients receiving bronchoscopy.

Technical progress in the chest TSCT examination has facilitated a more precise evaluation of existence and severity of emphysema and fibrosis. We recently demonstrated that the presence of TSCT-determined CPFE might predict worse postoperative and survival outcomes among patients with resected lung cancer [14]. In the previous study, more than 10% of patients with resected lung cancer were found to have fibrosis, 75% of whom had concurrent findings of emphysema. As a consequence, the prevalence of CPFE was 8.3% [14]. Our present data showed that 14.8% of the newly diagnosed lung cancer patients with bronchoscopy had fibrosis; 86.5% of these also had TSCT-determined emphysema. As a consequence, the prevalence of CPFE was 12.8%. Taken together, our data strengthens the theory that a TSCT examination at diagnosis for lung cancer successfully detects the presence of emphysema and fibrosis. In terms of the severity of emphysema in the patients with emphysema or CPFE, and in common with resected lung cancer cases [14], the proportion with emphysema grade 1 was significantly higher than those with emphysema grade 2 or 3. The previous data showed that the proportion of UIP pattern was 22.3% in patients with fibrosis and resected lung cancer (17 /76 cases) [14]. On the other hand, our present study demonstrated that the proportion of UIP pattern was 45.9% in the fibrosis group with newly diagnosed lung cancer (34/74 cases).

Whether the decision-making process involved in deciding therapeutic management options for lung cancer might be independently affected by the existence and severity of fibrosis in patients with lung cancer remains unclear. Therefore, we analyzed data from 343 patients with lung cancer at stage IA to IIIA because these patients are generally eligible for thoracic surgery with curative intent [6, 20, 25]. DLCO measurement is recommended for screening in the patients undergoing thoracic surgery, according to the European Respiratory Society (ERS) and the European Society of Thoracic Surgery (ESTS) clinical guideline [26]. DLCO is also a well-known survival predictor for patients with IPF and CPFE [27, 28]. Our data showed that DLCO measurement was not performed in more than 40% of surgical candidates missing thoracic surgery. Recent studies suggest that DLCO might be closely associated with the existence and extent of chest CT-detected emphysema and fibrosis [29, 30]. CPFE often involves upper lobe emphysema and lower lobe fibrosis [24]. The finding of TSCT-determined UIP pattern might affect survival in patients with CPFE [31]. DLCO measurement was performed for all five fibrosis patients, whereas more than 20% of CPFE patients did not have DLCO measurement (8/41 cases). Coexistence of TSCT-determined emphysema and fibrosis might affect the decision-making process for DLCO measurement with regards to postoperative residual lung function [32]. The previous study suggested that %DLCO, not TSCT-determined UIP pattern, did not have a significant and independent association with acute exacerbation incidence of interstitial lung diseases in patients with resected lung cancers [9]. Thus, another possibility might be due to insufficient evidence to show the importance of DLCO measurement for lung cancer treatment. Finally, we evaluated whether fibrosis severity might be an independent factor affecting decisions to propose thoracic surgery with curative intent. Our previous study showed that more severe airway obstruction (defined as GOLD grade 3), advanced clinical staging, and higher age, were independent factors associated with the decision to perform thoracic surgery [6]. Our present study strengthened the conclusion that these factors are critical and independent of the thoracic surgery decision. Furthermore, multivariate analysis showed that the TSCT-determined UIP pattern independently affects the decision-making process for thoracic surgery. Finding of honeycombing with or without traction bronchiectasis is critical for making a definite diagnosis of UIP [13]. Thus, coexistence of TSCT-determined emphysema and UIP pattern fibrosis might also affect the decision-making process for thoracic surgery with regards to postoperative residual lung function [14, 32].

This study was based on a retrospective analysis of data from consecutive newly diagnosed lung cancer patients who received bronchoscopy at a single institution. Although retrospective analysis might have several limitations to determine the hypothesis, these data-including 83.7% (499/596 patients) of all patients-are likely to minimize the possible contribution of selection bias due to different entry criteria for thoracic surgery [26]. We did not obtain pathologic confirmation of the fibrosis pattern from resected lung specimens. One reason is that the definition of CPFE was based on CT findings [24]. Another reason is that this study is aimed to evaluate the impact of TSCT-determined emphysema and fibrosis on the decision-making process for the treatment of lung cancer. Although the portion of lung resection might affect postoperative outcomes in resected lung cancer patients with fibrosis [9, 11], the portion of lung resection was not applied to our multivariate analysis. The decision for the portion of lung resection is usually made for surgical candidates who received preoperative spirometric assessment including DLCO [26]. Nevertheless, more than 40% of surgical candidates missing thoracic surgery did not receive DLCO measurement before the decision for the portion of lung resection.

More inclusive consideration for surgical resection with curative intent in lung cancer patients with chronic lung diseases should be required because limited surgical resections or nonsurgical therapeutic options might provide inferior survival compared with resection with curative intent. Recent studies show that multidisciplinary team management of lung cancer might improve survival outcomes [33, 34]. Furthermore, new therapeutic options for stable chronic lung diseases such as COPD/emphysema and pulmonary fibrosis have been utilized in the worldwide [15, 16]. This study provides a basis for further investigation into determining optimal management for lung cancer patients with chronic lung diseases through multidisciplinary team involvement with pulmonary specialists.

## Conclusion

The presence of TSCT-determined UIP pattern might independently affect the decision-making process for proposing thoracic surgery with curative intent.
